# A new bomb-combustion system for tritium extraction

**DOI:** 10.1007/s10967-017-5446-0

**Published:** 2017-09-08

**Authors:** Richard I. Marsh, Ian W. Croudace, Phillip E. Warwick, Natasha Cooper, Nadereh St-Amant

**Affiliations:** 1 GAU-Radioanalytical Laboratories, OES, National Oceanography Centre Southampton, University of Southampton, Southampton, SO14 3ZH UK; 2 Culham Centre for Fusion Energy, Abingdon, OX14 3DB UK; 3 Canadian Nuclear Safety Commission, Ottawa, ON K1P 5S9 Canada

**Keywords:** Tritium, Tritium extraction, Organically bound tritium, Bomb-combustion, Waste characterisation

## Abstract

Quantitative extraction of tritium from a sample matrix is critical to efficient measurement of the low-energy pure beta emitter. Oxidative pyrolysis using a tube furnace (Pyrolyser) has been adopted as an industry standard approach for the liberation of tritium (Warwick et al. in Anal Chim Acta 676:93–102, 2010) however pyrolysis of organic-rich materials can be problematic. Practically, the mass of organic rich sample combusted is typically limited to <1 g to minimise the possibility of incomplete combustion. This can have an impact on both the limit of detection that can be achieved and how representative the subsample is of the bulk material, particularly in the case of heterogeneous soft waste. Raddec International Ltd (Southampton, UK), in conjunction with GAU-Radioanalytical, has developed a new high-capacity oxygen combustion bomb (the Hyperbaric Oxidiser; HBO_2_) to address this challenge. The system is capable of quantitatively combusting samples of 20–30 g under an excess of oxygen, facilitating rapid extraction of total tritium from a wide range sample types.

## Introduction

Tritium (^3^H) is a low-energy pure beta emitter (*E*_max_ 18.6 keV) produced as a by-product of nuclear power plant operations, employed as a potential fuel in fusion reactors and utilised as a radiolabel in life science research. Direct measurement of ^3^H in solid and certain liquid samples is not possible as the weak beta emissions are rapidly attenuated by the sample matrix [1]. Liberation of ^3^H from the host matrix is therefore a critical stage in the analytical process. Numerous techniques have been developed to facilitate ^3^H extraction from a wide range of sample matrices including tube based combustion furnaces such as the Raddec Pyrolyser-6 trio [2, 3] and automated sample oxidisers such as the Perkin Elmer 307 and Zinsser Ox-501 [4]. Tube furnaces such as the Pyrolyser are highly flexible as they have been extensively tested, offer robust ^3^H extraction from a wide range of sample matrices [5] and can accommodate up to six samples per extraction. The principal limitation of such systems is that their capacity for organic-rich sample types must be limited to <1 g in order to minimise the possibility of pressure excursions and incomplete combustion [6]. Sample oxidisers are better suited to organic matrices but sample size is typically limited to between 0.5 and 1 g as such systems were originally developed for the analysis of radiolabelled biological material for which a high sample capacity was not required. Neither technique can be considered ideal for the analysis of organic-rich sample types and we can identify a number of scenarios where the ability to combust a much larger sample mass would be beneficial (Table 1).

**Table 1:** Analytical scenario where an approach capable of processing large, organic-rich samples matrices may be beneficial

^3^H analysis scenario	Analytical requirements/challenges
Environmental monitoring	Analysis of biota to assess potential food-chain transfer and dose to critical groups. Typically large sample sizes must be prepared in order to meet demanding limits of detection (LODs). This is particularly applicable to the assessment of organically bound tritium (OBT) [7, 8]
Nuclear decommissioning	Representative analysis of certain organic-rich orphan wastes such as plastics, elastomeric materials and oils that are difficult to process by other extraction techniques
Fusion reactor operations	Analysis of ^3^H contaminated soft wastes (e.g. gloves, coveralls, plastic sheeting, paper towel). Contamination may be heterogeneously distributed due to composition and large samples are required to ensure representative analysis

An alternative technique that has been successfully applied to the extraction of ^3^H from organic rich samples is oxygen-bomb combustion [9, 10]. Using this procedure, the sample is contained within a pressure vessel containing a significant excess of oxygen. Ignition results in rapid oxidation of the sample and the conversion of all hydrogen (and hence also ^3^H) present to H_2_O, which can subsequently be recovered for ^3^H analysis. The application of this approach has remained relatively limited however due to the lack of availability of commercial systems. Only the Parr model 1121 employed by Moghissi et al. [10] is readily available and sample mass is limited to a maximum of 10 g. Furthermore this system requires implementation of suitable ancillary equipment to enable the quantitative recovery of the resulting combustion water.

This paper outlines the evaluation of a recently developed oxygen-combustion bomb designed specifically to address these analytical requirements, the Raddec Hyperbaric Oxidiser, HBO_2_ [1, 11]. A summary of pre-production testing and two application case studies from current users of the system are described; environmental monitoring (Canadian Nuclear Safety Commission, Ottawa, Canada) and fusion reactor operational support (JET, Joint European Torus, CCFE, Didcot, UK).

## Materials and methods

### The hyperbaric oxidiser (HBO_2_)

**Fig. 1:**
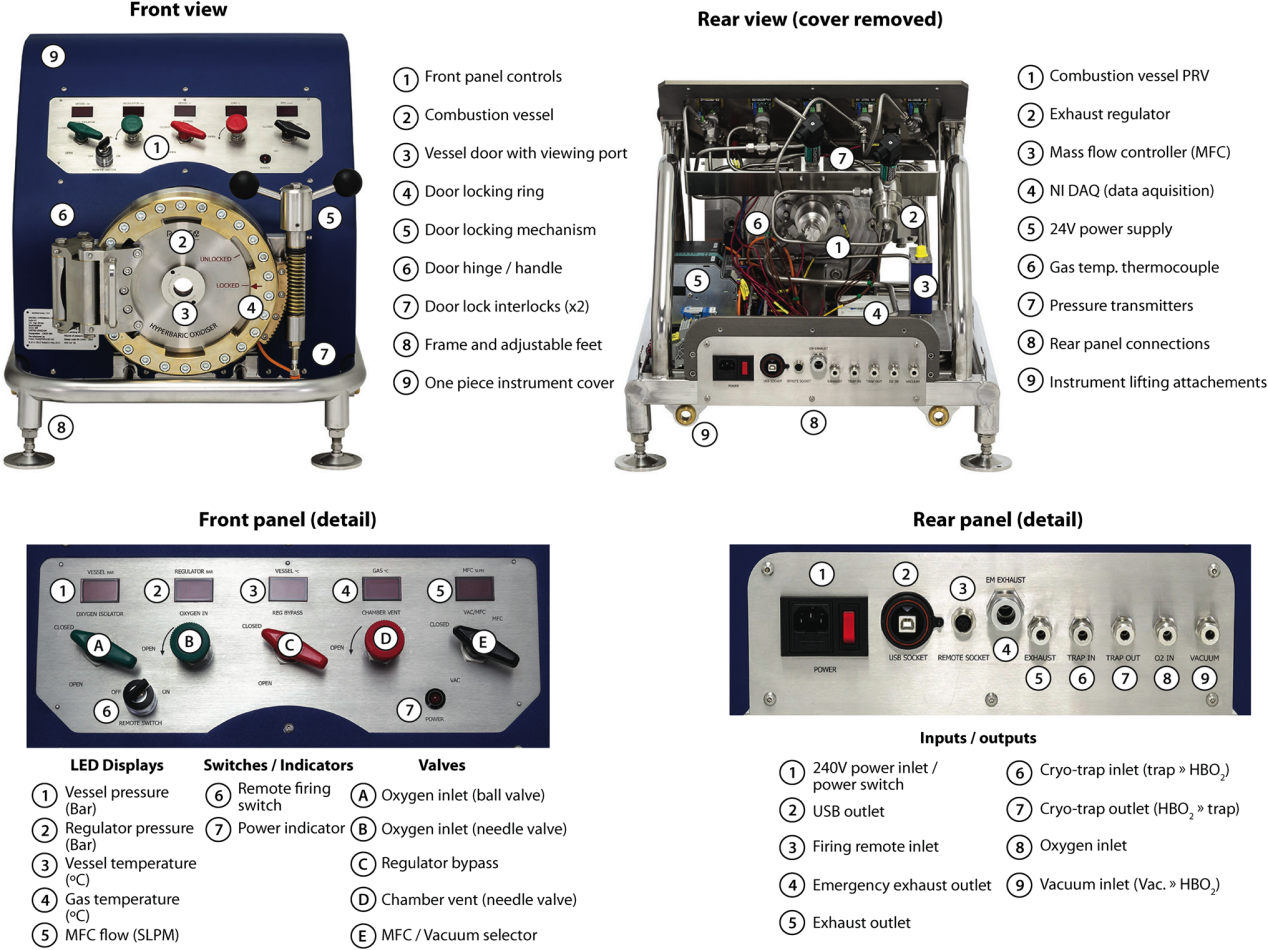
Key components of the HBO_2_

**Fig. 2:**
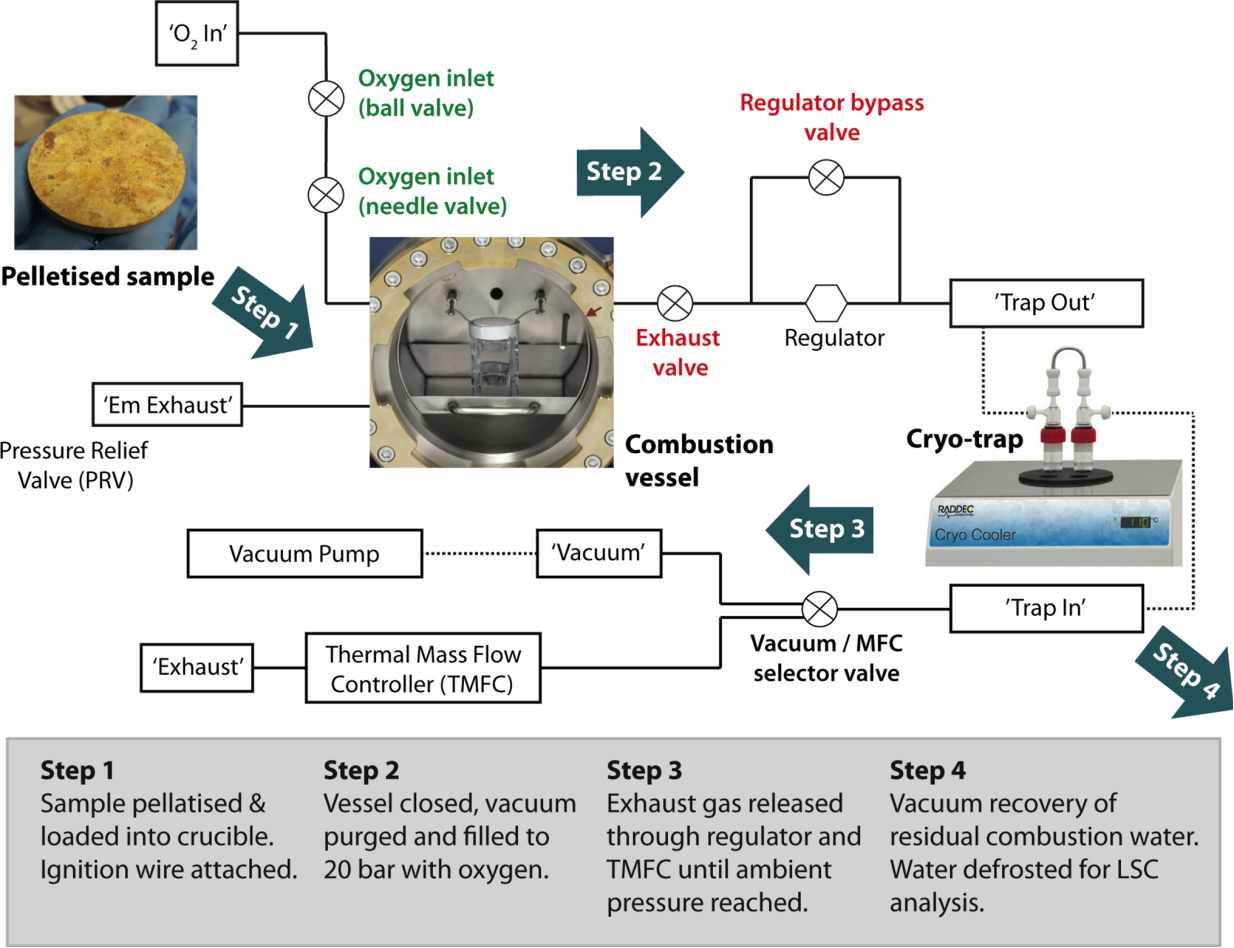
HBO_2_ flow schematic and combustion procedure

The HBO_2_ (Raddec International Ltd, Southampton, UK) comprises of a 5 litre high-strength pressure vessel machined from 316 grade stainless steel. A recent enhancement plans to use a more chemically-resistant Hastelloy™ that is more tolerant to HCl and nitric acid generated during oxidative decomposition. The vessel is supported within a stainless steel frame that also houses the combustion product recovery pipework, power (24 V) and monitoring systems (Fig. 1). The pressure vessel incorporates a resistance wire ignition system along with gas pressure and gas temperature sensors (K-type thermocouple) and a pressure-vessel temperature sensor. Samples are loaded into the vessel through a double-hinged door, which incorporates a borosilicate glass viewing window (Visilume, Glossop, UK) and tapered-lug door locking mechanism. The door closure mechanism incorporates dual safety interlocks to prevent ignition should the door not be properly secured prior to combustion. A removable stainless steel liner is installed to reduce wear and prolong pressure vessel life. Oxygen is introduced via gas inlet line governed by two independent valves located on the front panel (one needle type for fine metering, one ball for coarse control) and a check valve is installed to prevent backflow from the vessel. Exhaust gases leave the chamber via a 25 μm mesh stainless steel pre-filter and a 7 μm secondary, sintered filter (both removable for cleaning). Exhaust release is controlled via a needle valve on the front panel (valve ‘D’; Fig. 1) and can be diverted to a dual-borosilicate cryo-trap electrically-cooled to −110 °C via flexible PFA tubing for initial combustion water recovery or to a dry-scroll vacuum pump for recovery of residual water once ambient pressure is reached in the vessel. Exhaust gas flow is controlled by a single stage regulator and thermal mass flow controller (TMFC) (Fig. 2).

Vessel gas pressure, temperature and vessel body temperature data are measured during operation and output to digital displays on the front panel and by USB (‘2’ on rear panel detail; Fig. 1) to a computer via a NI DAQ and LabVIEW interface (National Instruments, Texas, USA). Regulator gas pressure and flow rate are also displayed. The combustion process is illustrated in Fig. 2. Solid samples may be combusted directly as can viscous liquids (e.g. oils). Powdered samples must be compressed into a pellet prior to combustion to prevent formation of an aerosol during filling/combustion that could result in an uncontrolled combustion. Typically a 40 mm diameter XRF press such as the HTP 40 (Herzog, Osnabruck, Germany) is used for pelleting, capable of compressing powdered samples to 200 kN.

### Pre-production evaluation

Initial evaluation of the system was completed through non-active testing and active commissioning. Non-active testing comprised of the combustion of cellulose ((C_6_H_10_O_5_)_n_) pellets of varying masses (5–25 g) and stoichiometric calculation of the theoretical combustion water yield. This was compared to the actual combustion water yield determined gravimetrically to infer recovery. Assessment of optimum vacuum recovery time and variation with sample mass was also completed by combustion of known quantities of cellulose. For active testing, 10 g cellulose pellets were spiked with a known activity of tritiated water (Amersham Bioscience, UK) and combusted. Aliquots of the combustion water were mixed with scintillation cocktail (Goldstar, Meridian Biotechnologies, UK) in 20 ml polyethylene vials (Meridian Biotechnologies, UK) and counted on a 1220 Quantulus Liquid scintillation Counter (LSC) (Wallac/Perkin Elmer, USA) to determine activity recovery. Samples of an organically bound ^3^H (OBT) reference material (^3^H-thymidine doped milk powder) were also pelletised with cellulose and combusted and the combustion water analysed in the same way.

### Environmental ^3^H analysis

Analysis of environmental samples was completed by Canadian Nuclear Safety Commission (CNSC), Ottawa, as part of routine regulatory surveillance in the vicinity of nuclear facilities known to handle ^3^H. Biota samples including fruit/berries, vegetables (beetroot, potatoes and carrot), milk and meat (beef, chicken and fish) were analysed for OBT content. All samples were dehydrated by freeze-drying and homogenised using a cutting/grinding mill (MF10, IKA, Germany). Approximately 10 g of each sample type was pelletised using a hydraulic press. Samples were combusted in the HBO_2_ under 20 bar of oxygen and the combustion water analysed directly or following distillation. Pelletised samples were also combusted using a Parr 1121 combustion bomb for comparison. Water was collected directly from the vessel and distilled prior to analysis. For all samples the combustion water was mixed with Ultima Gold LLT scintillation cocktail (Perkin Elmer, USA) and counted on a Tricarb TR3180 (Perkin Elmer, USA). Sample quench was evaluated using the tSIE parameter and corrected based on ‘in-house’ quench curves prepared using matrix-matched standards quenched with nitromethane.

### JET fusion reactor soft waste characterisation

Analysis of fusion reactor soft wastes was completed by Culham Centre for Fusion Energy (Didcot, UK) as part of their operational waste characterisation program. Samples of polyvinylchloride (PVC), fibreboard and ‘housekeeping waste’ (a heterogeneous mixture of nitrile gloves, cellulose paper/tissues, various plastics and cotton) were divided by hand (cutting) and pelletised using a hydraulic press. Microcrystaline cellulose powder (Sigma Aldrich, UK) was added to housekeeping samples as a binding agent and the effect of shrink wrapping pellets in non-PVC film prior to combustion was also investigated. Tritium memory effect of the HBO_2_ was assessed through subsequent combustion of blank cellulose pellets immediately following sample combustion and comparison of the total activity recovered with that of the sample. The combustion of blank cellulose pellets was also used to reduce the system memory between sample combustions. Subsamples of the combustion water (4 ml) were mixed with 16 ml of Ultima Gold scintillation cocktail (Perkin Elmer, USA) and counted on a Tricarb TR2810 (Perkin Elmer, USA). Constant quench was assumed provided that the tSIE parameter fell within a defined acceptable range. Subsamples of each sample type were also processed using a Pyrolyser-6 trio furnace (Raddec International, Southampton, UK) for comparison purposes. Samples were combusted using a 4-hour protocol under air and oxygen to a maximum temperature of 800 °C.

## Results

### Pre-production evaluation

**Fig. 3:**
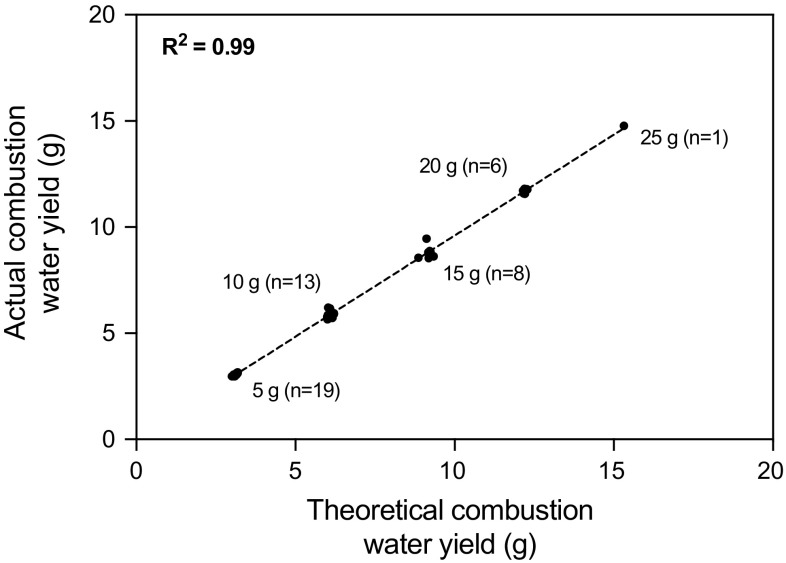
Actual versus theoretical combustion water yield for various masses of cellulose combusted using the HBO_2_

**Fig. 4:**
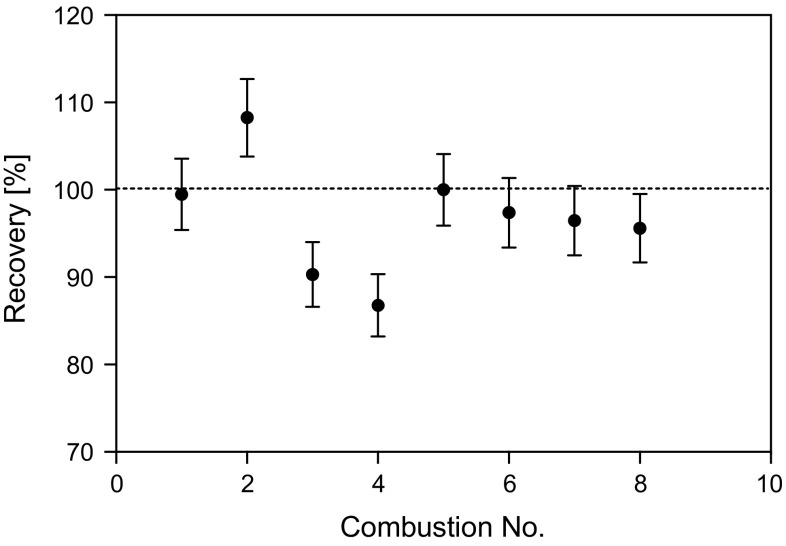
Activity based recovery for eight OBT combustions in the HBO_2_ (^3^H-thymidine doped milk powder; reference activity of 3.4 ± 1.2 Bq/g)

Complete recovery of combustion water resulting from 10 g cellulose pellets was achieved with vacuum pumping time of 10 min (based on a theoretical yield of 0.61 g of combustion water per gram of cellulose). The majority of combustion water was recovered in the first tube of the cryo-trap (91–95%) with minimal carry over to the second trap. Depending on the temperature of the combustion vessel a further 10 min per 10 g increase in sample mass was generally found to be sufficient for optimum combustion water recovery. The mass of combustion water recovered following the combustion of cellulose pellets from 5 to 25 g in mass showed close agreement with the theoretical mass (Fig. 3). For each test the distribution of liquid between the two cold traps remained constant within uncertainty (5–9% carried over to the second trap). A mean HTO recovery of 101 ± 3% (*k* = 2, *n* = 10) was determined with a mean counting efficiency of 23%. A mean OBT recovery of 97 ± 13% was achieved (*k* = 2, *n* = 8; Fig. 4) with a mean counting efficiency of 12%.

### Environmental ^3^H analysis

**Fig. 5:**
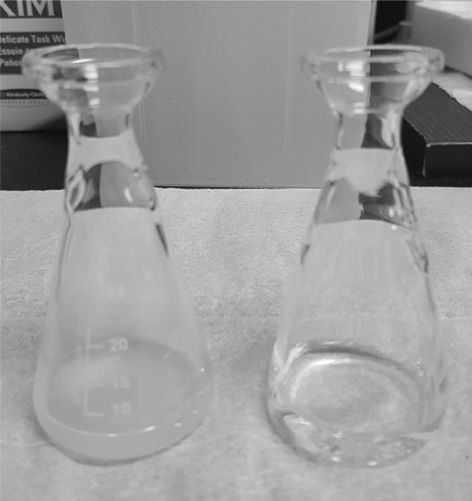
Comparison of combustion water obtained using the Parr 1121 (*left*) and HBO_2_ (*right*)

Successful combustion of all sample matrices was achieved in the HBO_2_, with minimal combustion residue and complete recovery of combustion water. The OBT activity measured in the biota samples ranged from 1.5 to 56 Bq/kg for both the Parr and HBO_2_ (in cases where samples were analysed by both systems results were below the LOD). Combustion of cellulose pellets between biota samples indicated no significant between-sample memory (results were LOD). Combustion water recovered from the HBO_2_ was noticeably clearer than water recovered directly from the Parr (Fig. 5). The LOD and counting efficiency for a typical sample are shown in Table 2 and a relative reduction in counting efficiency of up to 25% is apparent for combustion water from the Parr compared to that from the HBO_2_.

**Table 2:** LOD and counting efficiency data for the HBO_2_ and Parr 1121

Parameter	Hyperbaric oxidiser (HBO_2_)	Parr 1211 high capacity (1.85 L) bomb
Typical LOD (10 g sample)	2 Bq/kg	3 Bq/kg
Lower LOD (20–30 g sample)	1 Bq/kg (20 ml vial; potentially lower with 100 ml vial)	–
tSIE difference (ref. distilled water)	0–1%	12–24%

## ^3^H analysis in fusion reactor soft wastes

**Fig. 6:**
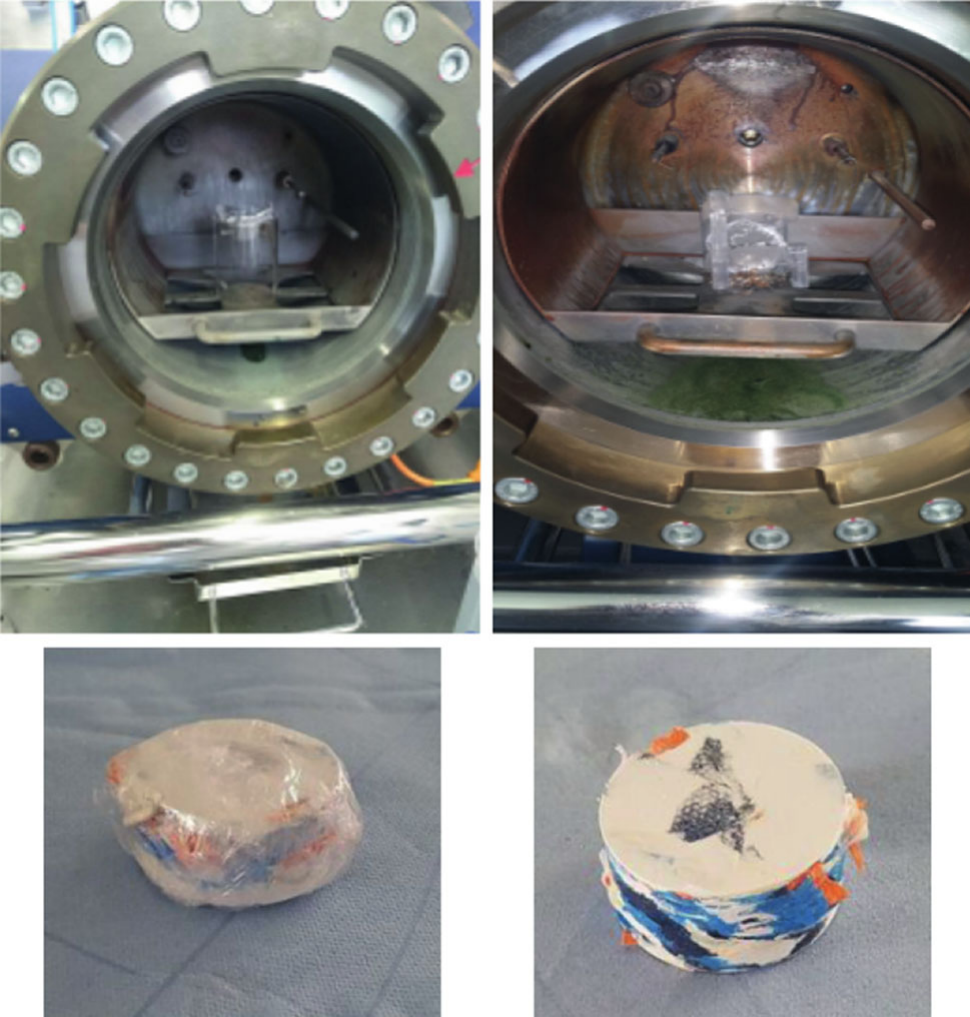
Comparison of residues after combustion of a mixed waste ‘housekeeping’ pellet with non-PVC film wrap (*left*) and without film wrap (*right*)

Fibreboard and PVC samples were combusted with minimal combustion residue and complete combustion water recovery. Combustion of ‘housekeeping’ samples initially resulted in elevated levels of combustion residue deposited inside the vessel liner, rear face and door. Incomplete combustion was reduced considerably by forming the pellet inside non-PVC wrapping film (Fig. 6). Activity data for samples of fibreboard, PVC and ‘housekeeping’ prepared using the HBO_2_ were comparable to measurements performed on sample prepared using the Pyrolyser furnace with the exception of the ‘housekeeping’ samples (Table 3). The ^3^H background or ‘memory effect’ following active sample combustion ranged from 0.3 to 1.3% (Table 3) for activity inventories of up to approximately 100 kBq.

**Table 3:** Fusion reactor soft waste activity data obtained using the HBO_2_ and Pyrolyser systems

Sample type	Hyperbaric oxidiser [Bq/g]	Pyrolyser [Bq/g]	HBO_2_ memory [%]
Fibreboard 1	11,300 ± 2000	11,000 ± 2000	0.62
Fibreboard 2	5900 ± 1000	8000 ± 2000	0.51
‘Housekeeping’ 1	600 ± 100	3100 ± 600	0.33
‘Housekeeping’ 2	80 ± 20	2700 ± 500	1.3
PVC	3000 ± 600	2600 ± 500	Not measured

The ^3^H ‘memory effect’ of the HBO_2_ system is also shown, measured in % of activity carried over from the active sample to a cellulose ‘blank’ combustion completed immediately afterwards

## Discussion

Pre-production evaluation of the performance of the HBO_2_ indicates that the system is capable of quantitatively combusting samples of 25 g. The upper limit for sample mass is dependent on sample type and maximum combustion pressure reached; for most matrices combustion of 30 g should be achievable. The combination of cryo-trapping, vacuum evacuation and an integrated gas handling system enables efficient combustion water recovery and hence tritium recoveries >95% for HTO and OBT. The former is indicative of efficient combustion water trapping where as the later validates both quantitative extraction and trapping. The variability associated with the OBT recovery is considered acceptable (±13%, *k* = 2) based on uncertainty of the standard reference value^1^ (34%, *k* = 2, *n* = 10). The significant difference in counting efficiencies observed between HTO and OBT samples is thought to be the result of acid formation during combustion of the milk matrix, which results in an increase in chemical quench during counting. By comparison, combustion of the cellulose matrix used for the HTO samples does not result in significant acid production (trace amounts of acid may be produced as a result of impurities in the cellulose and residual gasses present in the chamber from laboratory air). The author has used the addition of Na_2_CO_3_ to neutralise acids present in the combustion water and significantly improve counting efficiencies for organic rich samples (22–26% for marine biota samples [1]). Quantitative activity recovery is corroborated by a 1:1 ratio of theoretical versus actual combustion water yields for cellulose pellet combustions. Furthermore, assessment of vacuum pumping duration indicates that complete recovery can be achieved between 20 and 30 min for a 20 g sample depending on the vessel body temperature; enhanced evaporation occurs if successive combustions are completed over a short period [1].

A range of biota samples have been successfully combusted in the HBO_2_ and in all cases combustion residues were reduced when compared to similar combustions in the Parr system. This reduction is facilitated by the use of a special low-thermal mass crucible and quartz glass support, which minimises quench during combustion compared to a conventional metallic crucible. The vacuum recovery of combustion water combined with reduced combustion residue result in cleaner combustion water, which is less prone to colour and chemical quenching during LSC. It is also possible to recover a larger volume of combustion water for a comparable sample mass using the HBO_2_, resulting in an improvement in the LOD. Further LOD improvements are possible by maximizing sample mass when compared to other combustion bomb systems (up to the maximum volume of combustion water which can be counted per analysis – typically 8–10 ml for a 20 ml vial or potentially >15 ml for a 100 ml system). The most significant compromises when using the HBO_2_ are a slight increase in combustion time per sample and higher overall cost when compared to the Parr system. Comparison of operational experience with the HBO_2_ and Parr 1121 is given in Table 4.

**Table 4:** Operational comparison of HBO_2_ and Parr 1121 system for OBT extraction

Hyperbaric Oxidiser (HBO_2_)	Parr 1211 high capacity bomb
Maximum sample size of 25+ g	Maximum sample mass limited to 10 g
Sample combustion is visible via integrated viewing port	No viewing port for combustion monitoring
Provision of vacuum assisted combustion water recovery in easy to use cryo-traps.	Manual recovery of combustion water (complete recovery difficult)
Clear combustion water which can be analysed directly by LSC	Coloured combustion water which requires purification prior to analysis
Combustion time ~2 h per sample	Combustion time ~1 h per sample
Large system foot-print	Compact system foot-print
High initial system cost	Low initial system cost

The combustion of the diverse mixture of materials present in soft-waste streams generated by fusion reactor operations presents a significant challenge; oxidation of materials such as PVC and other plastics result in the production of strong acids and complex residues. Residues can be reduced by combining the sample with cellulose when pelleting and by wrapping the pellet in PVC-free wrapping film, which improves combustion efficiency and reduces combustion quench (note that the total activity recovered is not affected by the addition of cellulose hence no correction of the activity concentration is required when calculated back to the activity concentration of original sample). Damage to the combustion chamber is limited by the stainless steel liner and the removable internal filter limits ingress of combustion residue. Periodic cleaning of the gas handling system is achieved by flushing with a warm wash solution followed by distilled water rinse. Data for the waste materials tested show good agreement with that obtained by an established technique (Pyrolyser system) with the exception of the ‘housekeeping’ samples. The disparity between the data for this sample type is thought to be a result of the highly heterogeneous nature of this mixture and the associated sampling uncertainty. The HBO_2_ allows larger organic-rich samples to be processed in approximately 2 h per sample whereas a typical run on the Pyrolyser system has a duration of 4–6 h. Up to 6 samples may be combusted per run using the Pyrolyser however the mass of organic rich sample processed is typically 0.5–1 g (maximum 4–5 g) compared to the 10–30 g for the HBO_2_. When combusting organic-rich samples, particularly when approaching the maximum capacity, tube furnace systems such as the Pyrolyser are also prone to additional complications such as pressure excursions and increased combustion residues, which can impact tritium recovery.

In summary the HBO_2_ enables rapid and efficient recovery of tritium from a diverse range of organic rich sample matrices that can be problematic for other extraction approaches. The system is particularly applicable to the combustion of biota where low LODs are required for environmental monitoring or for the preparation of organic-rich materials originating from nuclear decommissioning or facilities with significant ^3^H inventories.

## Footnotes

1 Determined through replicate combustions of the standard using a Pyrolyser furnace as part of the characterisation process during standard preparation.
